# Distal vs. total pancreatectomy with celiac axis resection in patients with locally advanced/borderline resectable pancreatic carcinoma – a retrospective cohort study

**DOI:** 10.1186/s12893-026-04128-z

**Published:** 2026-08-12

**Authors:** Alexandros Chrysos, Iakovos Amygdalos, Roman Marius Eickhoff, Katharina Joechle, Franziska Meister, Lea Hitpass, Philipp Bruners, Sebastian Cammann, Oliver Beetz, Felix Oldhafer, Martin von Websky, Thomas Vogel, Florian Wolfgang Rudolf Vondran, Georg Wiltberger

**Affiliations:** 1 Department of General, Visceral, Paediatric and Transplant Surgery, University Hospital RWTH Aachen, Pauwelsstrasse 30, Aachen, 52074 Germany; 2 Department of Diagnostic and Interventional Radiology, University Hospital RWTH, Aachen, Germany

**Keywords:** Pancreas, pancreatectomy, Celiac trunk, Celiac axis resection, Embolization, Hepatic ischemia, Gastric ischemia

## Abstract

**Background:**

Distal pancreatectomy with celiac axis resection (DP-CAR) is an established procedure for the resection of celiac-trunk-infiltrating carcinomas of the pancreatic corpus or cauda. In contrast, total pancreatectomy with celiac axis resection (TP-CAR) remains a controversial procedure, which is sporadically mentioned in the literature. This study aimed to compare perioperative and survival outcomes following TP-CAR with those of the established DP-CAR operation.

**Methods:**

Between January 2010 and December 2023, 17 patients underwent DP-CAR and 8 patients TP-CAR in our institution. Pre-, intra-, postoperative, histopathological and survival data were compared between the two groups.

**Results:**

The TP-CAR cohort was associated with higher rates of major complications (DP-CAR: 2[12%] vs. TP-CAR: 4[50%], *p* = 0.037) and stomach ischemia (DP-CAR: 1[5.9%] vs. TP-CAR: 3[37.5%], *p* = 0.044). Length of stay in the intensive care unit was longer for the TP-CAR group (DP-CAR:1[1–1] vs. TP-CAR:18[8–27] days, *p* < 0.001). One patient in our study died postoperatively, belonging to the DP-CAR group. Median overall survival (DP-CAR:21[95% CI:16–26] vs. TP-CAR 14[95% CI:11.4–16.6] months, *p* = 0.355) and disease-free survival (DP-CAR: 12[95% CI:9.2–14.8] vs. TP-CAR: 10.5[5.4–15.6] months, *p* = 0.659) were higher in the DP-CAR group, without statistically significant difference in the log-rank test.

**Conclusions:**

Compared to DP-CAR, TP-CAR was associated with higher morbidity and longer stay in the intensive care unit, while mortality remained low in this cohort. Given the limited sample size and the absence of significant survival differences, TP-CAR may be considered in highly selected patients with good performance status and adequate tumour regression after neoadjuvant chemotherapy.

**Supplementary Information:**

The online version contains supplementary material available at 10.1186/s12893-026-04128-z.

## Background

In recent years, substantial advances in the oncologic and surgical treatment of pancreatic cancer have been made, however it remains the seventh leading cancer-related cause of death worldwide [1]. Early detection of pancreatic cancer still poses an oncologic challenge, as only 15–20% of pancreatic ductal adenocarcinoma (PDAC) are detected when primary surgical treatment is possible [2]. Surgical resection remains the only curative treatment and acceptable long-term survival can be achieved within multimodal therapy concepts. Thanks to recent advances in chemotherapy and implementation of rigorous neoadjuvant regimens, even borderline resectable pancreatic carcinoma (BRPC) and locally advanced pancreatic carcinomas (LAPC), which are initially deemed unresectable, can be resected with promising long-term survival [3]. Nevertheless, even after successful tumour downsizing, radical resection can be surgically challenging, since vascular involvement is present in most cases. A celiac trunk (CT) infiltrating carcinoma of the pancreatic tail or body is classified as T4 and a distal pancreatectomy with *en-bloc* celiac axis resection (DP-CAR) may be a potential option in selected cases. Celiac axis resection in combination with gastrectomy was first described by Appleby in 1953, as a radical oncologic resection technique for a celiac trunk infiltrating gastric carcinoma [4]. A modification of this technique, including an oncologic distal pancreatectomy, often foregoing the gastrectomy, was implemented in specialized centres and termed “modified” Appleby procedure [5]. The oncologic outcomes, morbidity and mortality of DP-CAR have been recently investigated in an increasing number of studies in high-volume centres, which reported encouraging results, similar to those of a conventional distal pancreatectomy (DP) [6, 7].

A key aspect of the procedure is ensuring an adequate collateral arterial blood flow to the liver via the pancreatic arcade and the gastroduodenal artery (GDA), in a regular anatomy of the hepatic artery. In certain cases, tumour infiltration extends to the GDA and proper hepatic artery (PHA), where a total pancreatectomy with celiac axis resection (TP-CAR) is mandatory. This surgical approach requires, in absence of an aberrant liver artery arising from the superior mesenteric artery (SMA), an arterial reconstruction of the PHA. Since postoperative pancreatic fistulae are irrelevant after total pancreatectomy, the arterial anastomosis and sufficient liver perfusion appear to be the most important factors regarding severe postoperative complications. To our knowledge, TP-CAR has been reported only in case reports or small retrospective cohorts [8, 9]. In contrast to DP-CAR, for which new data have emerged at an increasing rate in recent years, TP-CAR is rarely performed and is usually limited to high-volume centres with extensive experience in pancreatic surgery. The aim of this study is to compare surgical and oncologic outcomes between patients undergoing DP-CAR and TP-CAR to better understand their respective roles in the treatment of locally advanced pancreatic cancer.

## Methods

### Study design

**Fig. 1:**
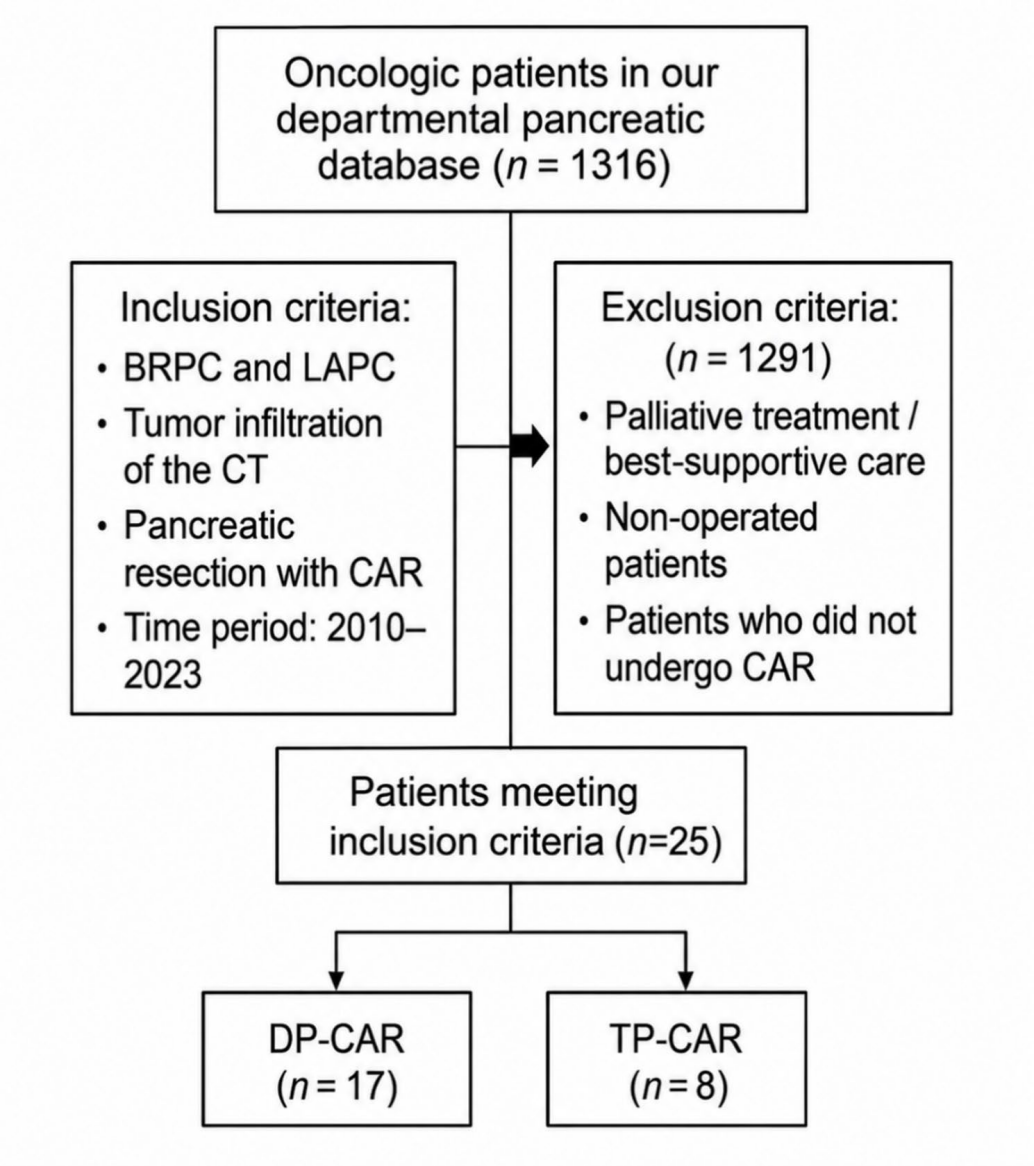
Flow Chart with patient inclusion/exclusion process

The search for eligible patients was conducted within our departmental pancreatic database, consisting of 1316 patients with carcinomas in any part of the pancreas. We included LAPC and BRPC patients with a tumorous infiltration of the CT, who underwent a pancreatic resection with a simultaneous celiac axis resection (CAR), between January 2010 and December 2023 in our department. The anatomical criteria for defining BRPC and LAPC were based on the National Comprehensive Cancer Network [3, 10, 11]. There were 25 patients fulfilling those criteria. Of those patients, 17 underwent a DP-CAR and 8 patients underwent a TP-CAR. The TP- and DP-CAR groups were compared and analysed according to preoperative, intraoperative, postoperative and survival parameters. The study was designed according to the Strengthening the Reporting of Observational Studies in Epidemiology (STROBE) guidelines for observational studies [12]. A flow chart illustrating the study’s inclusion process is shown in Fig. 1.

### Study aims

The aim of this study was to compare the well-established DP-CAR with the less frequently reported TP-CAR procedure based on postoperative clinical outcomes and survival. As the TP-CAR is an extension of the DP-CAR, it involves a more extensive resection and requires higher surgical expertise, due to the frequent need for arterial reconstruction of the PHA. Postoperative morbidity and overall survival (OS) were chosen as primary endpoints. Surgical margins and ischemic complications were this study’s secondary outcomes.

### Clinical parameters

Patients’ age, sex, body mass index (BMI), American Society of Anaesthesiologist’s (ASA) score, neoadjuvant chemotherapy, and preoperative embolization of the celiac trunk (PE-CT) were monitored preoperatively. Duration of surgery and type of resection were analysed as intraoperative parameters. Postoperative data concerning histological findings, hepatic or gastric ischemia, peak of liver transaminases in the first 3 days after surgery, 90 days morbidity and mortality after surgery were collected and analysed. Hepatic ischemia was defined as increased liver transaminases (Aspartate Aminotransferase [AST]) over 1000 Units per litre (U/L) and/or presence of perfusion-deficient liver regions detected in a postoperative computerized tomography scan. Gastric ischemia was defined as endoscopic findings of ischemic ulcers, requiring surgical or endoscopic treatment. Postoperative complications were stratified according to the Clavien-Dindo (CD) classification system as well as the comprehensive complication index (CCI) [13, 14]. Severe complications were defined as Clavien-Dindo equal or higher than 3b (CD ≥ 3b). The presence of postoperative pancreatic fistulae (POPF) was monitored in DP-CAR patients. POPF were classified according to the International Study Group on Pancreatic Fistula (ISGPF) definition [15]. Delayed gastric emptying and post-pancreatectomy haemorrhage were monitored in the study according to the International Study Group of Pancreatic Surgery (ISGPS) definitions [16, 17].

Resected tumours were examined in the Institute of Pathology of our Hospital and graded according to the 8th Edition of the Union for International Cancer Control (UICC) Tumour/Lymph node/Metastasis (TNM) classification. Data regarding local tumour infiltration, number of resected and positive lymph nodes, lymphovascular, vascular and perineural invasion, as well as resection margins (R) were obtained. The circumferential resection margin was characterized as positive (CRM+), when the minimal distance to the resected tumour was equal to or less than 1 mm and negative (CRM-) when the distance was larger than 1 mm. Accordingly R0 resections were further characterised as CRM positive or CRM negative.

### Preoperative embolization of the celiac trunk

Since the implementation of neoadjuvant chemotherapy in BRPC and LAPC, our clinic favoured a preoperative embolization of the celiac trunk (PE-CT) approximately 3–4 weeks prior to DP-CAR. This intervention’s intent was to enhance the collateral arterial blood flow to the liver from the SMA via the pancreatic arcade and the GDA, after occlusion of the common hepatic artery (CHA) through the embolization. Tumour in pancreatic body or tail, infiltration of the celiac trunk, tumour-downsizing after neoadjuvant chemotherapy, absence of new distant metastases and regular arterial liver anatomy were the criteria for a PE-CT in our clinic. Our hospital’s department of interventional radiology uses an AMPLATZER vascular plug to occlude the celiac trunk or the CHA. Collateral arterial perfusion of the liver through the GDA is then confirmed angiographically. Our institution’s PE-CT procedure has been described in detail by Zimmermann and colleagues [18].

### Operative technique

The pancreatic and celiac axis resection procedure was performed in our department by experienced hepatopancreatobiliary surgeons. All cases were discussed preoperatively in a multidisciplinary gastroenterological tumour board consisting of pancreatic surgeons, oncologists, gastroenterologists, radiologists and radiation therapists. Patients with locally advanced tumours of the pancreatic body or tail involving the celiac axis were considered candidates for DP-CAR. TP-CAR was planned for patients with similar tumour localisation but with further extension to the pancreatic head and/or involvement of the GDA or PHA, where preservation of the pancreatic head was not oncologically feasible. Following these anatomical considerations, patients were evaluated by the pancreatic surgery team regarding their performance score, fitness for surgery, nutritional status, comorbidities, tumour response to neoadjuvant chemotherapy and compliance. Patients fulfilling these criteria would be then scheduled for DP- or TP-CAR. In selected cases, a planned DP-CAR was converted intraoperatively to TP-CAR when frozen section analysis demonstrated more extensive tumour involvement than anticipated preoperatively.

The DP-CAR procedure has been extensively described in literature [19]. An artery first approach is routinely applied in our department. After excluding distant metastases, we begin with a dissection of the CHA, SMA, GDA and portal vein (PV). Tumour infiltration of the GDA and SMA must be excluded through palpation and frozen section. In patients without a PE-CT, the collateral arterial blood flow in the PHA will be measured with a doppler probe after clamping the CHA. After confirmation of adequate liver perfusion, we proceed with the pancreatic resection.

The DP part of the procedure is carried out in our department as a radical antegrade/posterior modular pancreaticosplenectomy (RAMPS) [20]. The left gastric artery (LGA), the CHA proximal of the GDA, and then the celiac trunk are ligated and divided. The right gastroepiploic and gastric arteries are spared, when possible, to reduce gastric complications. The LGA will also be spared, if it emerges separately from the abdominal aorta. After division of the pancreas at the portomesenteric axis we evaluate gastric perfusion. According to the extent of the gastric tissue ischemic demarcation, we proceed with a partial, subtotal or total resection of the stomach. A standardized lymphadenectomy is conducted as defined by ISGPS [21]. Pancreas, spleen, and parts of the stomach are removed *en-bloc* with the celiac axis. A frozen section of the pancreatic resection margin is then evaluated by an experienced pathologist. Excellent communication between surgeon and pathologist is essential at this operative step. Negative resection margins confirm sufficient oncologic resection. However, in case of positive medial resection margins, an extended pancreatic resection lateral to the portomesenteric axis is necessary to achieve complete tumour clearance. Consequently, conversion of the planned DP-CAR procedure to a TP-CAR procedure becomes necessary. As mentioned above, in 8 patients of our study TP-CAR was necessary. Of those 8 patients, two were initially planned as DP-CAR and had to be converted to a TP-CAR intraoperatively, due to microscopic tumour invasion of the pancreatic head, detected through frozen section.

The TP-CAR procedure is performed in our department as an extension of the DP-CAR. The dissection of the pancreatic tail and body, as well as that of the celiac axis will be performed similar to the DP-CAR procedure. The dissection of the head of the pancreas, common bile duct and the duodenum will be performed following the principles of a Whipple procedure, including the standardized lymphadenectomy in this region. One major aspect of the TP-CAR is gastric resection. Its extent is evaluated intraoperatively, based on the stomach’s perfusion and signs of gastric ischemia. Gastric perfusion is assessed based on the macroscopic appearance of the gastric wall, including colour, capillary refill and bleeding from the resection margins. A total gastrectomy can be avoided, if the LGA can be spared as noted previously. In that case, a 4/5 gastrectomy is favoured by our department. After complete resection, we strive to provide the pathologist with an intact *en-bloc* specimen containing the pancreas, spleen, duodenum, bile duct, celiac axis, regional lymph nodes and a major part of the stomach.

**Fig. 2:**
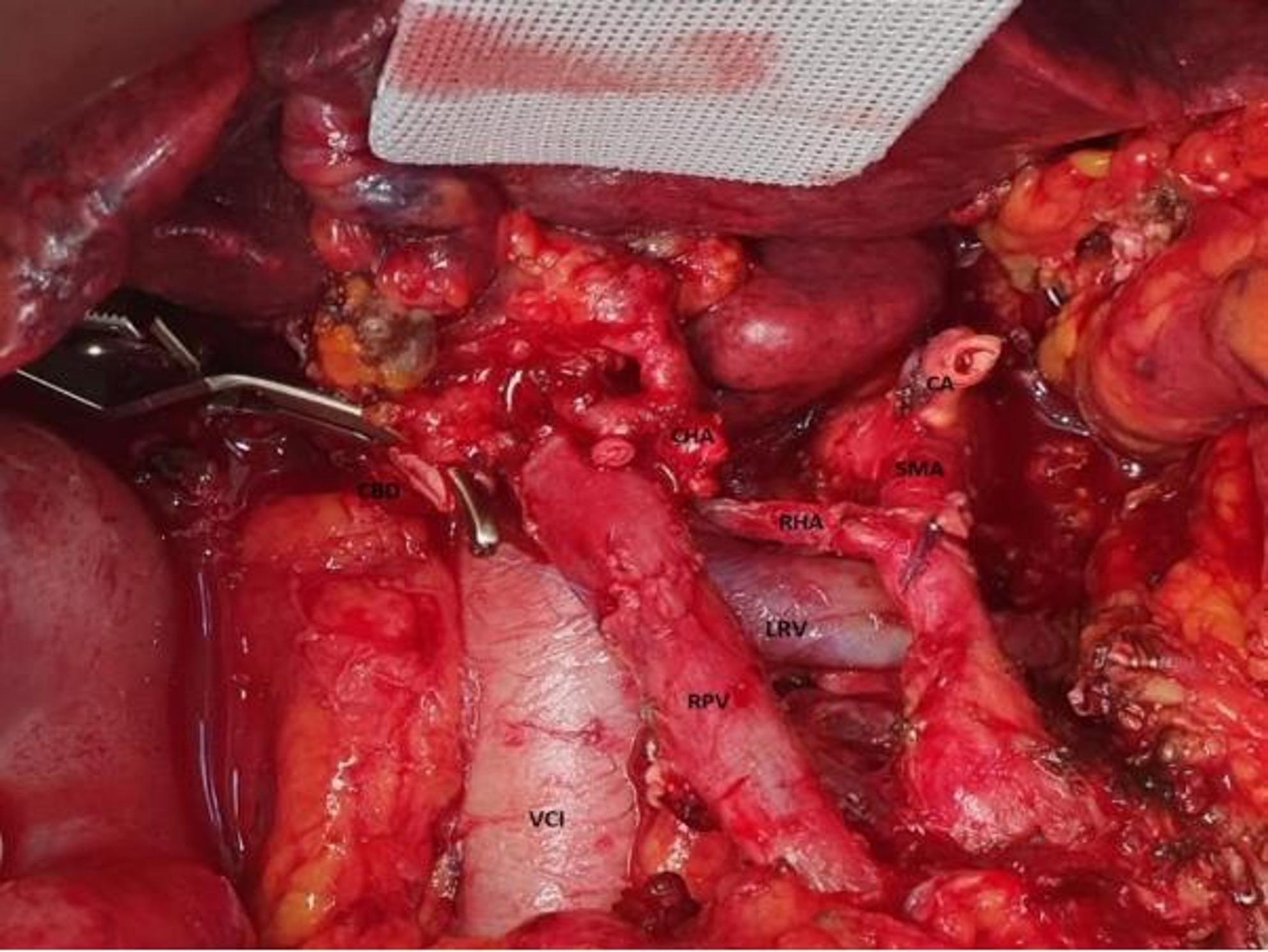
Intraoperative view after TP-CAR in a patient with an aberrant right hepatic artery. Legend: CBD, Common bile duct; CHA, Common hepatic artery; CA, Celiac axis (resected); SMA, Superior mesenteric artery; RHA, right hepatic artery; LRV, left renal vein; RPV, reconstructed portal vein; VCI, Vena cava inferior

As mentioned before, since resection of pancreatic head in a TP-CAR involves the GDA, CHA and pancreatic arterial arcade, the arterial hepatic inflow through the PHA is absent in a normal arterial anatomy. Therefore, arterial reconstruction is required to restore arterial blood supply to the liver. Our department favours arterial anastomosis of the PHA to the abdominal aorta. This anastomosis is performed either with an autologous graft, using the resected splenic artery, or with a polytetrafluoroethylene (PTFE) prosthetic graft, using 7/0 non-absorbable running sutures. The splenic artery is preferred whenever it is free of tumour infiltration and provides sufficient length and calibre for a tension-free anastomosis. PTFE grafts were used when these conditions were not met. Adequate hepatic arterial perfusion is confirmed with doppler ultrasound both intraoperatively and postoperatively. Postoperative monitoring of the hepatic arterial perfusion is continued twice daily for five days. CT imaging is performed when vascular complications are suspected. According to our institutional protocol, all patients undergoing arterial reconstruction receive 100 mg of aspirin daily lifelong. In case of venous reconstruction, therapeutic heparin was administered. An intraoperative view following TP-CAR is shown in Fig. 2.

### Follow-up

Patients received routine follow-up, including abdominal imaging, tumour marker assessment, and physical exams. Suspected tumour recurrence prompted additional imaging/biopsy for confirmation. Follow-up data were monitored from January 2010 until the 31st of December 2024, which was defined as end of follow-up. One patient was lost to follow-up. Overall survival (OS) was defined as survival in months until end of follow-up or death of the patient. Disease free survival (DFS) was defined as time in months until the date of local or distant recurrence. Finally, cancer-specific survival (CSS) was defined as survival period, measured in months, until end of follow-up or cancer-associated death of a patient. Patients who died from non-oncologic occurrences were not included in that analysis.

### Statistical analysis

Data were reported as medians and interquartile range (IQR, given as 1st-3rd quartiles) for continuous variables or absolute and relative frequencies for categorical and ordinal variables. Group comparisons were carried out using the Mann-Whitney U test, the Chi-square test or Fisher’s exact test as applicable. Survival data are depicted as medians with 95% confidence intervals (CI). Kaplan-Meier analysis and the log-rank test were used to compare differences in OS, DFS and CSS. All *p*-values < 0.05 were considered statistically significant. Statistical analysis was performed using SPSS Statistics v28 (IBM Corp., Armonk, NY, USA) and graphs were generated using Prism v8.0 (GraphPad Software, La Jolla, CA, USA).

### Ethics approval and consent to participate

The study was conducted under ethical approval of the Independent Ethics Committee of the RWTH Aachen Faculty of Medicine (EK 25–040) and in accordance with the current version of the Declaration of Helsinki, the Declaration of Istanbul, and good clinical practice guidelines (ICHGCP). Informed consent was waived due to the retrospective study design and collection of readily available clinical data.

## Results

A total of 25 patients underwent pancreatectomy with CAR in the study period. Of those patients, 15 (58%) were female and median age was 67 (60–72) years. Median ASA Score was 3 (2–3). Preoperatively, 18 (72%) patients received neoadjuvant or induction treatment (all from 2018 onwards). Regarding applied regimens, 12 received FOLFIRINOX and 4 received Gemcitabine/Abraxane (nab-paclitaxel). Detailed regimen information was unavailable for the remaining patients. DP-CAR was performed on 17 (68%) patients, and 8 (32%) patients underwent an angiographic embolization of the celiac trunk or CHA preoperatively. Intraoperatively, eight patients (32%) received a simultaneous gastrectomy, and median operational duration was 300 (214–493) minutes. Regarding the histopathological status, a total of 17 (68%) patients had nodal positive status, the median number of resected lymph nodes was 27 (19–36) and the median number of infiltrated lymph nodes was 2 (0–4). An R0 resection was achieved in 16 (64%) patients. Half of those patients (*n* = 8) had negative circumferential resection margins (CRM-). Postoperatively, a total of 3 (12%) patients suffered from liver ischemia and 4 (16%) patients exhibited signs of gastric ischemia. These three patients who developed hepatic ischemia demonstrated elevated transaminase values as well as hepatic hypoperfusion in the CT imaging. The median peak of liver-transaminases in the first three postoperative days was 230 (65–729) U/L in the general cohort. POPF grade B was detected in 47% of DP-CAR patients and no Grade C fistula was detected in the study population. In 3 (12%) patients post-pancreatectomy haemorrhage was detected and two of those patients suffered from a grade C haemorrhage. The PHA was the source of haemorrhage in both cases. In the first case, the bleeding was arrested angiographically with a stent implantation, and the second patient’s haemorrhage was managed surgically. Of the 22 patients who did not receive a total gastrectomy intraoperatively, 3 (13.6%) of those suffered from delayed gastric emptying. Regarding postoperative complications, median CCI was 37.1 (28.4–51.7) and major complications (CD ≥ 3b) were detected in 6 (24%) patients. Median length of stay in the intensive care unit (ICU-LOS) was 1 (1–11) day and LOS in the standard care ward was 24 (14.5–34.5) days. The study population’s 90-day mortality was 4% (*n* = 1). Postoperatively, 14 (56%) patients received adjuvant chemotherapy in the form of FOLFIRINOX or Gemcitabine with or without Abraxane. Patients who did not receive adjuvant chemotherapy either underwent surveillance or were considered unfit for systemic treatment because of postoperative complications or poor postoperative recovery. One patient declined chemotherapy, despite recommendation. Median OS was 19 (95% CI: 12.3–23.7) months, median DFS was 10.5 months (95% CI: 8.7–12.3) and median CSS was 20 (95% CI: 16–24) months. The 1- and 2-year survival rates were 68% and 26%, respectively. Preoperative, intraoperative, postoperative histological and survival parameters are depicted in Table 1.

**Table 1:** Perioperative, histological, postoperative, and oncological characteristics of the study population and subgroups

Parameters	All patients (*n* = 25)	TP – CAR (*n* = 8)	DP – CAR (*n* = 17)	*p*=
Gender *Female*	15 (60%)	4 (50%)	11 (64.7%)	0.324
Age [y]	67 (60–72)	68 (59–77)	66 (60.5–72.5)	0.887
BMI [kg/m²]	24 (22–27)	22.6 (21.7–28)	24.3 (21.8–26)	0.932
ASA	3 (2–3)	2 (2–3)	3 (2–3)	0.344
Neoadjuvant CTx	18 (72%)	8 (100%)	10 (58.8%)	**0.032**
Preoperative Embolization	8 (32%)	1 (12.5%)	7 (41.2%)	0.152
OP-Duration [min]	300 (226–491)	543 (488–665)	266.5 (211–351)	**< 0.001**
Intraoperative Gastrectomy *Total* *Subtotal/Partial*	8 (32%) 3 (12%) 5 (20%)	6 (75%) 1 (12.5%) 5 (62.5%)	2 (11.8%) 2 (11.8%) 0	**0.002**
UICC 8th Edition, Grade of Differentiation				
*G1*	0	0	0	
*G2*	9 (64%)	1 (33.3%)	8 (72.7%)	0.205
*G3*	5 (36%)	2 (66.7%)	3 (27.3%)	
Tumour size *T1/T2* *T3* *T4*	6 (24%) 16 (64%) 3 (12%)	2 (25%) 6 (75%) 0	4 (23.5%) 10 (58.8%) 3 (17.6%)	0.235
Nodal status *N1* *N2/N3*	12 (48%) 5 (20%)	5 (62.5%) 3 (37.5%)	7 (41.2%) 2 (11.8%)	0.319 0.133
Resected LN	27 (19–36)	32 (24–41)	25 (16-32.5)	0.086
Positive LN	2 (0–4)	3 (1–7)	2 (0-3.5)	0.097
Lymphovascular invasion (L1) Vascular invasion (V1) Perineural invasion (Pn1)	7 (30%) 11 (44%) 20 (83%)	4 (50%) 5 (62.5%) 8 (100%)	3 (20%) 6 (35.3%) 12 (75%)	0.136 0.201 0.121
Residual Tumour status *R0* *R1*	16 (64%) 9 (36%)	4 (50%) 4 (50%)	12 (70.6%) 5 (29.4%)	0.316
CRM+	8/16 (50%)	3/4 (75%)	5/12 (41.7%)	0.438
Postoperative hepatic ischemia	3 (12%)	2 (25%)	1 (6%)	0.170
Transaminase-Peak 3d postop. [U/L]	230 (65–729)	1145 (175–2280)	141 (58.5–448)	**0.019**
Postoperative gastric Ischemia	4 (16%)	3 (37.5%)	1 (6%)	**0.044**
PPH	3 (12%)	1 (14.3%)	2 (12%)	0.659
DGE	3/22 (13.6%)	1 (20%)	2/13 (16%)	0.337
CCI	37.1 (28.4–51.7)	60.6 (36.9–90)	30.8 (26.9–47.2)	**0.023**
CD ≥ 3b	6 (24%)	4 (50%)	2 (11.8%)	**0.037**
Mortality	1 (4%)	0	1 (6%)	0.484
ICU – LOS [d]	1 (1–11)	18 (8–27)	1 (1–1)	**< 0.001**
SC - LOS [d]	24 (14.5–34.5)	37 (16–77)	23 (13-27.5)	0.097
Adjuvant CTx	14 (56%)	3 (38%)	11 (65%)	0.201
1-year Survival	16 (66.7%)	4 (50%)	12 (75%)	0.221
2-years Survival	6/23 (26%)	1/7 (14.3%)	5/16 (31.3%)	0.394
Median OS [months]	19 (95% CI: 12.3–23.7)	14 (95% CI: 11.4–16.6)	21 (95% CI: 16–26)	0.355
Median DFS [months]	10.5 (95% CI: 8.7–12.3)	10.5 (95% CI: 5.4–15.6)	12 (95% CI: 9.2–14.8)	0.659
Median CSS [months]	20 (95% CI: 16–24)	17 (95% CI: 11–23)	21 (95% CI: 17–25)	0.715

*DP-CAR* distal pancreatectomy with celiac axis resection, *TP-CAR* total pancreatectomy with celiac axis resection, (*y*) years, (*d*) day, *BMI* Body Mass Index, *ASA* American Society of Anaesthesiologists’ score, *OP* Operational; min, Minutes, *UICC* Union for International Cancer control, *LN* Lymph nodes, *CRM+* positive circumferential resection margins, *U/L* Units per litre, Postop. postoperative, *POPF* postoperative pancreatic fistula, *PPH* post-pancreatectomy haemorrhage, *DGE* Delayed gastric emptying, *CCI* Comprehensive Complication Index, *CD ≥ 3b* Clavien-Dindo Complications Grade ≥ 3b, *ICU* intensive care unit, *LOS* Length of Stay, *SC* Standard Care, *OS* Overall survival, *DFS* Disease Free Survival, *CSS* Cancer Specific Survival

### Non-embolized vs. embolized cohorts

In the first subgroup analysis, we compared patients who received a preoperative embolization of the celiac trunk or CHA, against those who did not. The embolized group consisted of 8 (32%) patients and the second group of 17 (68%) patients. The demographic, preoperative and postoperative characteristics didn’t vary significantly in the two groups. The embolized cohort of our study suffered from neither hepatic nor gastric ischemia after surgery. On the contrary, three patients (18%) suffered from liver ischemia and four (24%) from gastric ischemia in the non-embolized group, although these differences were not statistically significant. The parameters of the two subgroups are summarized in Supplementary Table 1.

### TP-CAR vs. DP-CAR

After the embolization analysis, we conducted a second subgroup analysis comparing TP-CAR with DP-CAR patients, based on the same parameters. The DP-CAR group consisted of 17 (68%) patients and the TP-CAR group of 8 patients (32%). Patients’ demographic features didn’t vary significantly between the two groups. All TP-CAR patients received neoadjuvant chemotherapy, in contrast to the DP-CAR cohort where 10 (58.8%) patients were treated prior to surgery (*p* = 0.032). In 7 (41.2%) patients of the DP-CAR group a PE-CT was performed. The duration of surgery was significantly longer in the TP-CAR group (TP: 543 [488–665] min vs. DP: 266.5 [211–351] min, *p* < 0.001). A simultaneous gastrectomy was more frequently performed in the TP-CAR group (TP: 6 [75%] vs. DP: 2 [11.8%], *p* = 0.002).

Specifically in the TP-CAR group, after resection of the pancreas and the GDA, an arterial reconstruction of the PHA was required in 5 patients with normal arterial anatomy. An arterial anastomosis of the PHA with the infrarenal abdominal aorta was performed using a PTFE prosthetic graft in 3 patients, and a reconstructed autologous artery was used as an arterial graft in 2 patients. In the 3 patients, where a right aberrant arterial circulation of the liver from the SMA existed, there was no need for arterial reconstruction. A PV-reconstruction was performed in 7 patients, where the PV showed microscopic signs of tumour infiltration.

**Fig. 3:**
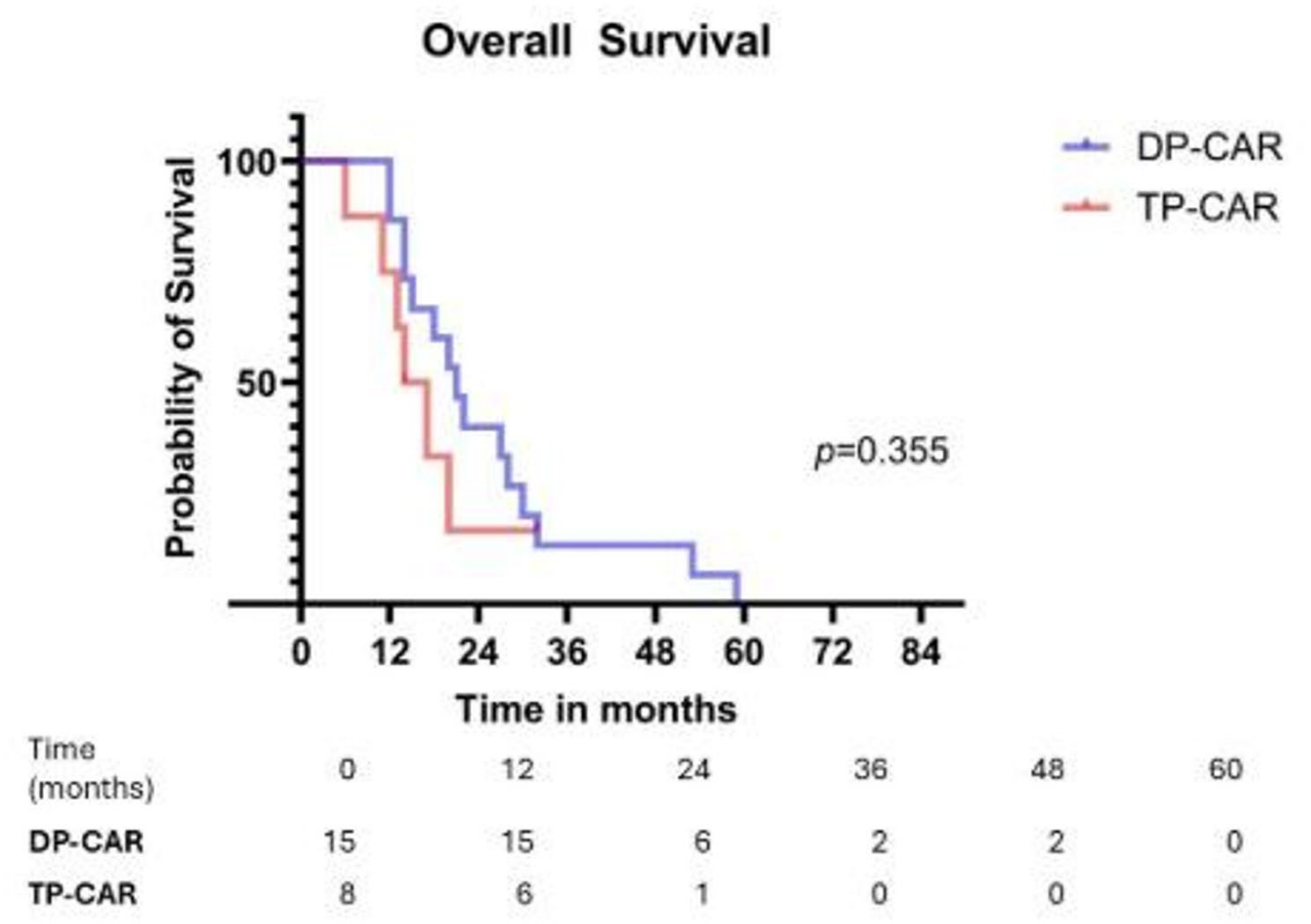
Overall Survival (OS) for patients with TP- and DP-CAR

**Fig. 4:**
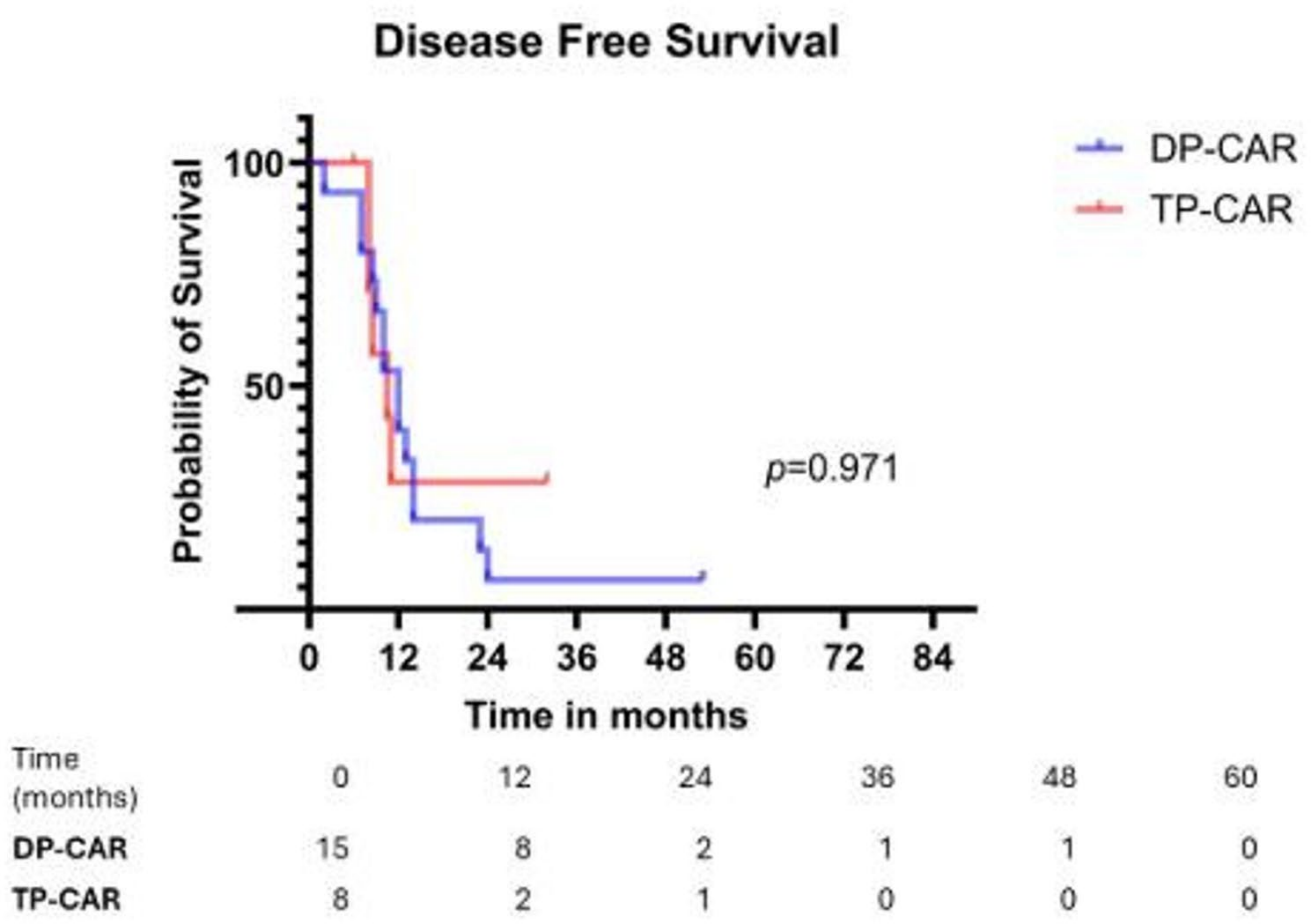
Disease Free Survival (DFS) for patients with TP- and DP-CAR

The two groups didn’t show any statistically significant differences in the histological findings. Noteworthy is the rate of R0 resections, in the DP-CAR group 12 (70.6%) patients had a complete resection, in contrast to the TP-CAR group where a R0 resection was observed in 4 (50%) patients (*p* = 0.316). Regarding the postoperative parameters, the TP-CAR group was associated with a higher median postoperative peak of transaminases in the first 3 postoperative days (TP: 1145 [175–2280] U/L vs. DP: 141 [58.5–448] U/L, *p* = 0.019). Hepatic ischemia was more frequent in that group too, but without statistical significance (TP: 2 [25%] vs. 1 [5.9%], *p* = 0.170). However, only one patient developed clinically relevant hepatic ischemia with subsequent liver failure. This patient belonged to the DP-CAR cohort and died during the postoperative course. In the TP-CAR group, gastric ischemia was more prevalent, as 3 (37.5%) patients suffered from it, whereas in the DP-CAR group only 1 (5.9%) patient demonstrated endoscopic signs of impaired gastric perfusion (*p* = 0.044). One of these four patients was treated conservatively, but the rest required surgical intervention. Median CCI (TP: 60.6 [36.9–90] vs. DP: 30.8 [26.9–47.2], *p* = 0.023) and major complications (CD ≥ 3b TP: 4 [50%] vs. DP:2 [11.8%], *p* = 0.037) were higher in the TP-CAR group. ICU-LOS was significantly higher in the TP group (TP: 18 [8–27] days vs. DP: 1 [1–1] day, *p* < 0.001), however the LOS in the standard care ward did not differ significantly between the two groups (TP: 37 [16–77] days vs. DP: 23 [13-27.5] days, *p* = 0.097). No patient of the TP-CAR group died within 90 days after surgery. In the present study 11 (65%) DP-CAR and 3 (38%) TP-CAR patients received adjuvant chemotherapy, the difference did not reach statistical significance (*p* = 0.201). A detailed comparison of the study parameters between the two cohorts is portrayed in Table 1  and data regarding adjuvant treatment are shown in Supplementary Table 2. Median follow-up time was 15.5 (95% CI: 7.3–24.5) months with a follow-up completeness rate of 96%. Median OS and DFS were 14 (95% CI: 11.4–16.6) and 10.5 (95% CI: 5.4–15.6) months respectively in the TP-CAR group. The DP-CAR was associated with higher survival medians, OS at 21 (95% CI: 16–26, *p* = 0.355) and DFS at 12 (95% CI: 9.2–14.8, *p* = 0.659) months, lacking significant difference. The Kaplan-Meier Curves for OS and DFS are shown in Figs. 3 and 4.

## Discussion

This study demonstrates that although TP-CAR is a highly invasive procedure, often requiring arterial and vascular reconstruction to ensure adequate hepatic perfusion, reasonable short- and long-term results can be achieved in carefully selected patients. The arterial anastomosis is the most sensitive aspect of the intraoperative and postoperative course, necessitating increased clinical vigilance regarding early detection of ischemic complications. This becomes evident in our group comparison, as the TP-CAR group was associated with higher rates of gastric (38%) and hepatic ischemia (25%) compared to the DP-CAR group (liver: 6%, stomach: 6%). Klompmaker and colleagues reported hepatic ischemia rates of 17.7% and gastric ischemia rates of 4.4% in a large pan-European retrospective DP-CAR study [7]. Ischemic complications constitute decisive factors of postoperative outcome in pancreatic surgery with arterial resections. Unfortunately, definitions of hepatic and gastric ischemia remain heterogenous in literature, which renders comparison of different cohorts difficult. In a Japanese single-centre study by Nakamura et al., a higher ischemic gastropathy at 28.8% is reported in DP-CAR patients, but only 6.3% suffered from a severe gastropathy with perforation [22]. They also reported that liver infarction was as low as 6.3%. Noteworthy is that all patients routinely underwent a preoperative embolization of the CHA. A similar preoperative strategy was described in the study of Storkholm et al. to enhance the collateral arterial blood supply to the liver and stomach [23]. Hepatic ischemia in that study was 5% and the authors recommended preoperative embolization after careful evaluation of the arterial anatomy. In our study, ischemic complications were observed only in patients without a preoperative embolization of the CHA. Although this finding may suggest a protective effect, due to the small sample size and lack of statistical significance no definitive conclusions can be drawn. To our knowledge, the existing data in the literature are obtained mostly from retrospective studies with small to medium cohorts, resulting in a sporadic and often experience-based implementation of the PE-CT or -CHA. However, not all DP-CAR patients benefit equally from a PE-CT, therefore a generalized implementation of the embolization for every patient planned for DP-CAR is not advisable. A careful preoperative examination of every patient’s arterial anatomy is required. A GDA of small calibre and absence of an aberrant right HA from the SMA are anatomic factors which would warrant a preoperative angiography and possibly an embolization of the CHA in DP-CAR patients. A PE-CT should not be considered if the CHA is transposed from the SMA.

In TP-CAR patients, the GDA, and the pancreatic arcade are resected with the head of the pancreas, therefore, there is no collateral blood flow to the liver in regular CHA anatomy. Thus, in almost all TP-CAR cases arterial reconstruction with autologous or synthetic graft is necessary, to ensure sufficient arterial hepatic perfusion. Different reconstruction techniques are described in the literature. A reconstruction of the hepatic artery using the middle colic artery was used by Suzuki et al. [24]. Other authors reported the use of the splenic artery [25] or the saphenous vein [26] as auto-graft. We found the splenic artery technique promising for TP-CAR patients, with respect to the vessel’s diameter and regionality. An arterial anastomosis can be avoided in the special case of a hepatic artery originating from the SMA. Adequate arterial flow to the liver should be confirmed with ultrasound after resection.

Some authors suggest that a subtotal gastrectomy in TP-CAR is possible, if an extensive dissection of the gastroesophageal junction can be avoided, especially since the stomach offers nutritional advantages [8]. Considering our TP-CAR cohort’s higher rate of stomach ischemia, a more aggressive approach regarding total gastrectomy may be justified. A partial gastrectomy should only be considered, if the LGA stems separately from the aorta and can be preserved. Moreover, gastric vein congestion (GVC) is another cause of major morbidity, in the form of mucosal erosions, ulcers, bleeding and gastroenterostomy leakage. In a retrospective study from a high-volume institution, GVC was associated with significantly higher 90-day mortality (7.4%) [27]. To avoid GVC in DP-CAR, the gastric coronary vein should be maintained, as all the left-sided venous drainage is removed after resection of the spleen [27]. The subphrenic veins around the gastroesophageal junction are responsible for the venous outflow in TP-CAR, which puts a larger stomach remnant at risk of GVC.

As a result of the higher rate of ischemic complications, the TP-CAR group exhibited higher major morbidity (50%) compared to the DP-CAR group (11.8%). Since these two procedures are not interchangeable, the purpose of this comparison was not to suggest that one procedure should be preferred over the other, but rather to place the outcomes of the rarely reported TP-CAR procedure into the context of the established DP-CAR operation. Given the scarcity of published TP-CAR series, DP-CAR represents in our opinion the most appropriate reference cohort for perioperative and oncologic outcomes after celiac axis resection. The rate of major morbidity in the literature’s larger retrospective DP-CAR cohorts lies between 25-67.7% [7, 23, 28]. Data on TP with arterial resection are scarce. In a large single-centre retrospective study that examined TP patients with arterial resection, a surgical morbidity of 59.4% was described [29]. Our findings are similar, with half of the TP-CAR cohort population suffering from major complications. TP-CAR is a longer procedure, with more extensive dissection and the benefit of the absent POPF is offset by postoperative pancreoprivic diabetes. Patients that undergo TP-CAR require aggressive postoperative surveillance for the early detection of severe complications. In our department, we have implemented a twice-daily assessment of liver perfusion with Doppler ultrasound alongside laboratory parameters for the first five postoperative days in patients undergoing DP- or TP-CAR.

As mentioned previously, TP-CAR and DP-CAR are surgically demanding procedures and should be performed in high-volume centres, since morbidity and mortality appear to be lower in these institutions [30]. These were defined by Klompmaker and colleagues, as centres that perform at least one DP-CAR per year [30]. Our department falls into that category, as at least one DP-CAR has been performed yearly, while half the study population was operated between January 2020 to April 2022, resulting in a major leap concerning our institution’s experience. Loos et al. reported similar rising numbers of DP-CAR in recent years [31]. In their cohort study, the benefits of higher expertise are evident, as 90-day mortality declined from 25% to 3.6% with the increase of centre experience over time [31]. DP-CAR mortality ranges from 5% to 16.4% in larger retrospective cohorts [7, 22]. In our study, 90-day mortality remains low, as only one patient died 22 days after surgery from multiorgan failure after hepatic and gastric ischemia, resulting in a rate of 4%. As mentioned above, the TP-CAR cohort had higher rates of postoperative ischemia and major morbidity but with absent mortality.

Complete tumour resection and tumour-free resection margins (R0) play a major role in oncologic outcomes after pancreatic resection, and an R0 resection was achieved in 62.5% of the study cohort [32]. Retrospective cohort studies show results with an R0 rate ranging from 54.6–76% [7, 23, 31]. Probably the highest R0-Status in DP-CAR patients was reported by Nakamura and colleagues, where the R0 rate was 92.5% [22]. This high R0 percentage was accompanied by equally high median OS for DP-CAR patients, at 30.9 months. The authors point out the importance of the celiac axis resection with a complete retroperitoneal dissection, to achieve high R0 rates in carcinomas of the pancreatic body that tend to progress dorsally. The DP-CAR cohort showed higher rates of negative resection margins (70.6%) in our study. The larger resection area of the TP-CAR procedure and discontinuous posterior tumour growth makes frozen section more demanding than DP-CAR, with possibly higher rate of false negative resection margins. In our own experience, intraoperative false negative results occur in up to 12% of cases with arterial tumour involvement [32]. Regarding TNM status of the TP-CAR patients, none of them exhibited pT4 tumours, which may be attributed to tumour downsizing after intensive neoadjuvant chemotherapy.

Similarly to the larger DP-CAR cohorts in the literature, where OS ranges between 18 and 30 months, the DP-CAR cohort’s median OS was 21 months [7, 22, 31]. A lower median OS at 14 months and median CSS at 17 months were observed in the TP-CAR cohort, but the difference did not reach statistical significance. One apparent reason for that difference could be the lower R0 rate of the TP-CAR patients, since negative resection margin is a major factor that determines survival in patients undergoing pancreatectomies with arterial resection [32]. Another aetiology of this group’s poorer outcome is the long-term morbidity. Two patients died of non-oncologic causes, myocardial infarction and pneumonic sepsis, 6 and 11 months after surgery, respectively. These patients did not show recurrence. These findings should, however, be interpreted with caution given the limited sample size of both cohorts. The small sample size of both cohorts, particularly the TP-CAR group, substantially limits statistical power and increases the risk of a Type II error. Therefore, the absence of statistically significant differences in survival outcomes should not be interpreted as evidence of equivalence between the two procedures.

The alternative to the TP-CAR procedure would be palliative chemotherapy. With recent chemotherapy regimens, FOLFIRINOX and Gemcitabine/nab-paclitaxel, a median OS of 11.1 and 8.5 months respectively was achieved in patients with unresectable or metastatic pancreatic carcinoma, who undergo specialized palliative treatment [33, 34]. FOLFIRINOX was associated with higher OS in these patients, but also with higher toxicity and long-term treatment is rarely tolerated. Current evidence shows that LAPC and BRPC patients with adequate tumour response after chemotherapy should undergo oncologic resection, as this treatment strategy is associated with longer overall survival [35, 36]. This resection has acquired the term “conversion surgery” in the literature and appears to be a promising treatment in these patients [37]. These findings suggest that TP-CAR may represent a potential treatment option in highly selected patients who remain eligible for surgical resection after neoadjuvant therapy and in whom DP-CAR is not oncologically feasible. Nevertheless, candidates for such a highly invasive procedure should have good performance status and would probably benefit from intensive prehabilitation, to improve their preoperative condition.

The retrospective design and the small sample size are the main limitations of this study. These two factors reduce the statistical power, particularly in the TP-CAR cohort and restrict the generalizability of the results. Our data portray our department’s experience and operative technique over a long period of time. Over that period there have been changes in pancreatic oncology, such as the introduction of neoadjuvant chemotherapy for BRPC or periadventitial arterial dissection. All TP-CAR patients received neoadjuvant chemotherapy, whereas 58.8% of DP-CAR patients did, introducing treatment imbalance. On the other hand, 65% of DP-CAR patients and 38% of the TP-CAR patients received adjuvant chemotherapy. These differences reflect the evolution of pancreatic cancer treatment strategies during the study period and introduce potential confounding. Consequently, differences in postoperative and oncologic outcomes between the two groups cannot be attributed solely to the extent of surgical resection. Complete data regarding administration and completion of adjuvant treatment were not available for all patients because part of the oncological treatment was performed at external institutions and could not be fully included in the analysis. The more advanced disease biology and surgical extent in TP-CAR may confound comparisons of morbidity and survival. Our study’s selection bias should be mentioned, since a DP-CAR or a TP-CAR was only performed in highly selected patients with good condition and high-performance status. Our two cohorts show some heterogeneity concerning surgical radicality of surgery, as total pancreatectomy is a much more radical procedure. Moreover, the annual number of CAR procedures increased in our department over time, and experience could have played a role. Another missing parameter from our study would be the patients’ quality of life postoperatively. Major operations, especially with resection of the stomach and pancreoprivic diabetes, have a major impact on patients’ lives.

## Conclusions

TP-CAR is a highly invasive procedure associated with considerable morbidity, but it may offer a potentially curative treatment option for carefully selected patients with locally advanced and borderline resectable pancreatic carcinoma. Due to its high surgical complexity, TP-CAR should be reserved for high-volume hepatobiliary pancreatic centres and for patients demonstrating good tumour response after neoadjuvant chemotherapy. Careful evaluation of each patient’s arterial anatomy is essential for efficient reconstruction of the hepatic arterial perfusion.

## Supplementary Information

Supplementary Material 1.

Supplementary Material 2.

## Data Availability

The datasets used and/or analyzed during the current study are available from the corresponding author on reasonable request.

## Abbreviations

ASA: American Society of Anaesthesiologist’s score
BRPC: Borderline resectable pancreatic carcinoma
CAR: Celiac axis resection
CCI: Comprehensive Complication Index
CD: Clavien-Dindo classification
CHA: Common hepatic artery
CRM: Circumferential resection margin
CSS: Cancer specific survival
DFS: Disease Free Survival
DP: Distal pancreatectomy
DP-CAR: Distal pancreatectomy with celiac axis resection
GDA: Gastroduodenal artery
GVC: Gastric vein congestion
ICU: Intensive Care Unit
ISGPF: International Study Group on Pancreatic Fistula
LAPC: Locally advanced pancreatic carcinoma
LGA: Left gastric artery
LOS: Length of Stay
PHA: Proper hepatic artery
PE-CT: Preoperative embolization of the celiac trunk
POPF: Postoperative pancreatic fistulae
PTFE: Polytetrafluoroethylene
PV: Portal vein
SMA: Superior mesenteric artery
TP: Total pancreatectomy
TP-CAR: Total pancreatectomy with celiac axis resection
U/L: Units per litre
