# Supplementary material for: Distal vs. total pancreatectomy with celiac axis resection in patients with locally advanced/borderline resectable pancreatic carcinoma – a retrospective cohort study
Source: BMC Surg. 2026 Aug 12;26:537. doi: 10.1186/s12893-026-04128-z (PMC13465456; doi:10.1186/s12893-026-04128-z)
Supplement: Supplementary file 1 [file 12893_2026_4128_MOESM1_ESM.docx]

**Supplementary Table 1:** Comparison of patients with and without preoperative embolization of the coeliac trunk or common hepatic artery.

| Parameters | Embolized  N=8 (32%) | Non-Embolized  N=17 (68%) | *p=* |
| --- | --- | --- | --- |
| Gender  *Female*  *Male* | 4 (50%)  4 (50%) | 11 (64.7%)  6 (35.3%) | 0.484 |
| Age (y) | 64 (56-72) | 68 (61-73) | 0.511 |
| BMI | 24 (22-25) | 24 (21-28) | 0.669 |
| ASA | 3 (2-3) | 3 (2-3) | 1.0 |
| Neoadjuvant CTx | 5 (63%) | 13 (76.5%) | 0.468 |
| OP-Duration (min) | 214 (195-455) | 334 (264-502) | 0.087 |
| DP-CAR | 7 (87.5%) | 10 (59%) | 0.152 |
| Intraoperative Gastrectomy  *Total*  *Subtotal/Partial* | 1 (12.5%)  1 (12.5%)  0 | 7 (41%)  2 (12%)  5 (29%) | 0.152 |
| UICC 8th Edition,  Grade of Differentiation  *G1*  *G2*  *G3* | 0  4 (67%)  2 (33%) | 0  5 (63%)  3 (37%) | 0.872 |
| Tumour size  *T1/T2*  *T3*  *T4* | 1(12.5%)  7 (88%)  0 | 5 (29%)  9 (53%)  3 (18%) | 0.356  0.093  0.205 |
| Nodal status  *N1*  *N2/N3* | 5 (63%)  0 | 7 (41%)  5 (29%) | 0.319  0.086 |
| Resected LN | 29 (17-35) | 25 (21-37) | 0.887 |
| Positive LN | 2 (0-3) | 2 (0-5) | 0.669 |
| Lymphovascular invasion (L1)  Vascular invasion (V1)  Perineural invasion (Pn1) | 2 (33%)  3 (38%)  6 (86%) | 5 (29%)  8 (47%)  14 (82%) | 0.858  0.653  0.841 |
| Residual Tumour status  *R0*  *R1* | 6 (75%)  2 (25%) | 10 (59%)  7 (41%) | 0.432 |
| CRM+ | 2/6 (33%) | 6/10 (50%) | 0.302 |
| Transaminase-Peak postop. (U/l) | 275 (95-983) | 142 (65-719) | 0.842 |
| Postop. Liver ischemia | 0 (0%) | 3 (17.6%) | 0.205 |
| Postop.  Gastric Ischemia | 0 (0%) | 4 (23.5%) | 0.134 |
| POPF No Fist. Or Bioch. Leak | 2/7 (29%) | 7/10 (70%) | 0.092 |
| POPF Grade B | 5/7 (71%) | 3/10 (30%) | 0.092 |
| POPF Grade C | 0 | 0 |  |
| CCI | 28 (24-46) | 40.5 (30-68) | 0.075 |
| CD ≥ 3b | 2 (25%) | 4 (23.5%) | 0.936 |
| Mortality | 0 (0%) | 1 (6%) | 0.484 |
| SC - LOS (d) | 26 (17-33) | 23.5 (14-34) | 0.787 |

DP-CAR, distal pancreatectomy with celiac axis resection; (y), years; (d), day; BMI, Body Mass Index; ASA, American society of anaesthesiologists’ score; OP, Operative; min, Minutes; UICC, Union for International Cancer control; LN, Lymph nodes; CRM+, positive circumferential resection margins; U/L, Units per litre; Postop. , postoperative; PPH, post-pancreatectomy haemorrhage; DGE, Delayed gastric emptying; CCI, Comprehensive Complication Index; CD ≥ 3b, Clavien Dindo Complications Grade ≥ 3b; LOS, Length of Stay; SC, Standard Care; OS, Overall survival; DFS, Disease Free Survival
