# Supplementary material for: Distal vs. total pancreatectomy with celiac axis resection in patients with locally advanced/borderline resectable pancreatic carcinoma – a retrospective cohort study
Source: BMC Surg. 2026 Aug 12;26:537. doi: 10.1186/s12893-026-04128-z (PMC13465456; doi:10.1186/s12893-026-04128-z)
Supplement: Supplementary file 2 [file 12893_2026_4128_MOESM2_ESM.docx]

**Supplementary Table 2:** Adjuvant chemotherapy administration after DP-CAR and TP-CAR

| Parameters | All (n=25) | TP-CAR (n=8) | DP-CAR (n=17) |
| --- | --- | --- | --- |
| Adjuvant CTx | 14 (56%) | 3 (38%) | 11 (65%) |
| *FOLFIRINOX*  *Gemcitabine ± Abraxane (nab-paclitaxel)* | 7 (50%)  7 (50%) | 1 (33%)  2 (67%) | 6 (54%)  5 (46%) |
| No Adjuvant CTx | 11 (44%) | 5 (63%) | 6 (35%) |
| *Surveillance*  *Poor PS*  *Refusal**  *Death*** | 4 (36%)  5 (46%)  1 (9%)  1 (9%) | 1 (20%)  3 (60%)  1 (20%)  0 | 3 (50%)  2 (33%)  0  1 (17%) |

DP-CAR, distal pancreatectomy with celiac axis resection; TP-CAR, total pancreatectomy with celiac axis resection; PS, performance status.

*Patient declined chemotherapy despite recommendation. **Death occurred before adjuvant chemotherapy could be initiated.
